# Preliminary exploration of theory and practice training of 5G ultrasonic remote consultation in grassroot hospitals

**DOI:** 10.1186/s12913-022-08221-w

**Published:** 2022-06-24

**Authors:** Ceng Wang, Yi Zheng, Cui Xiong, Litao Sun, Jing Wang

**Affiliations:** 1 Health Management Center, Department of Ultrasound Medicine, Zhejiang Provincial People’s Hospital, Affiliated People’s Hospital, Hangzhou Medical College, Hangzhou, 310014 Zhejiang China; 2 Health Management Center, Department of Nursing, Zhejiang Provincial People’s Hospital, Affiliated People’s Hospital, Hangzhou Medical College, Hangzhou, 310014 Zhejiang China

**Keywords:** Grassroots hospitals, 5G ultrasound, Apparatus

## Abstract

**Background:**

With the rapid development of science and technology, telemedicine diagnosis and treatment systems have gradually attracted increased attention and applications.5G ultrasound is an important branch of telemedicine, connecting grassroots hospitals at one end and provincal hospitals at the other, which provides remote guidance to grassroots doctors for ultrasound examination and image diagnosis. It is convenient for villagers obtaining diagnosis and advice from provincial ultrasound experts, saving time and economic costs, as well as benefiting from high-quality ultrasound medical resources. In this study, taishun County community grassroot hospitals were selected as the pilot study of 5G ultrasound application, to explore the effectiveness of their theory and practice, and gradually improve the remote ultrasound diagnosis and treatment standards, so as to improve their quality of grassroots hospitals and benefit grassroots people.

**Methods:**

This is a descriptive study. The Provincal Hospital will conduct ultrasonic theory and practice training for grassroot hospitals. The training subjects included 43 doctors in grassroots hospitals who were willing to carry out ultrasound examinations. Theories, skills training scores and trainees' questionnaires on teaching content were collected and analyzed. After passing theoretical and practical training, they will conduct ultrasound examinations in their respective communities and collect relevant cases. There are 148 cases thus far for analysis. It mainly included the type of disease, whether the patient was out-patient or inpatient, frequency of ultrasound visits in recent 5 years, and follow-up treatment measures.

**Results:**

It mainly included three aspects: (1) Through theoretical and practical training, the ultrasonic diagnosis level of grassroot doctors was significantly improved. The difference in scores between the two practical trainings was statistically significant. (2) Forty-three questionnaires were sent out, feedback from trainees was very high. Most of them was very satisfied with our training. The total score of the questionnaire was 10, and 97.67% of them score more than 8. (3) In total, there were 148 remote consultation cases, including 67 males and 81 females, who were aged 21 to 101 years old (62.40 ± 15.73).mainly abdominal ultrasound, and typical cases involve fatty liver, hepatic cyst, gallbladder stone, kidney stone and so on. We analyzed case data and provided follow-up treatment recommendations.

**Conclusion:**

As a “visual apparatus”, 5G ultrasound can be routinely carried out in grassroot hospitals, which can provide mutual benefit between doctors and patients and comprehensively promote healthy villages.

## Background

China has increased the development of primary medical and health facilities, and the supporting hardware equipment has kept pace with the demand for diagnosis and treatment. However, there is still a shortage of primary medical personnel, especially experienced sonographers. Some clinicians are part-time sonographers. In the face of a large population, the ultrasound examination task is demanding, and the pressure is huge.

Due to a lack of systematic theoretical knowledge training and clinical practice operations, it is easy to miss diagnoses and misdiagnose [1], and the quality of ultrasound examination can be greatly reduced, which can have a negative impact on grassroots hospitals (smaller health centres in towns and communities). Many patients, even after seeing common diseases in a grassroots hospital, still question the diagnosis result and then go to a tertiary hospital for repeated examination, which requires time, increases the economic burden, and wastes medical resources. In the past, the methods to improve the level of ultrasonic diagnosis and treatment at the grassroots level mainly included grassroots doctors going to provincial tertiary hospitals for further study. The provincial hospital has basic level lectures and formulates ultrasonic inspection specifications, and the quality control center conducts regular inspections. However, due to a lack of human resources in grassroots hospitals, the study time is short, and progress is slow. Provincial hospitals teach and provide lectures in grassroots hospitals, but they are only at the theoretical level, and the scope is limited. The formulation of ultrasonic diagnosis and treatment standards can improve the level of ultrasonic diagnosis and treatment at the grassroots level to a certain extent, but it has little effect on the development of grassroots ultrasonic medicine at the early stage.

With the rapid development of science and technology, telemedicine diagnosis and treatment systems have gradually attracted increased attention and applications. 5G ultrasound is an important branch of telemedicine, connecting grassroots hospitals at one end and provincial hospitals at the other, which provides remote guidance to grassroots doctors for ultrasound examination and image diagnosis [2]. Following its comprehensive application, villagers can directly realize face-to-face remote consultation with expert doctors in community hospitals instead of rushing to tertiary hospitals and waiting in line for registration. 5G ultrasound is convenient for obtaining diagnosis and advice from provincial ultrasound experts, saving time and economic costs, as well as benefiting from high-quality ultrasound medical resources. As early as 2017, starting from Pengbu Health Center, Jianggan District, Hangzhou, our hospital officially carried out ultrasonic remote consultation projects with many grassroots hospitals, such as Haining and Chun 'an. Taishun County is located in a remote mountainous area in southern Zhejiang Province, where transportation is not convenient and medical resources are weak. Therefore, In this study, taishun County community grassroots hospitals were selected as the pilot study of 5G ultrasound application, to explore the effectiveness of their theory and practice, and gradually improve the remote ultrasound diagnosis and treatment standards, so as to improve their quality of grassroots hospitals and benefit grassroots people.

## Methods

This is a descriptive study. The guidance hospital for 5G ultrasound for this study was Zhejiang Provincial People’s Hospital, and the grassroots hospital was Taishun County People's Hospital and 12 affiliated community health hospitals. The training took place in October 2020. The content of theory and skills training is shown in Table 1 and Table 2. The training subjects included 43 doctors in grassroots hospitals who were willing to carry out ultrasound examinations. Theories, skills training scores and trainees’ questionnaires on teaching content were collected and analyzed. After passing theoretical and practical training, they will conduct ultrasound examinations in their respective communities and collect relevant cases (mainly, long-term residents in the community and cases with limited mobility). There are 148 cases thus far for analysis. It mainly included the type of disease, whether the patient was out-patient or inpatient, frequency of ultrasound visits in recent 5 years, and follow-up treatment measures All experimental protocols were approved by the Ethics Committee of Zhejiang Provincial People's Hospital. Informed consent was obtained from all subjects.

**Table 1:** Theoretical training

	Content	Knowledge points to master
1	Principle and application of ultrasound medicine	The application range of ultrasound
2	Standard image for conventional ultrasound applications	Remember the anatomy of the standard section
3	Ultrasonographic assessment of trauma	Key points of ultrasound assessment of trauma
4	Ultrasonographic assessment of acute abdomen	Key points of ultrasound assessment of acute abdomen
5	Ultrasonographic assessment of shock/hypoxemia	Key points of ultrasound assessment of shock/hypoxemia

**Table 2:** Practical training

	Content	Knowledge points to master
1	Routine section of the liver	a. Sagittal section of the left lobe of the liver through the abdominal aorta, b. Oblique section of the right subcostal side through the first porta of the liver, c. Oblique section of the costal margin through the second porta of the liver, etc
2	Longitudinal section of the gallbladder	Measure the maximum diameter of the gallbladder
3	Section of spleen	Measuring the thickness of the spleen (through the hilum of the spleen)
4	Section of both kidneys	Measure the maximum diameter of the kidney (through the hilum of the kidney)
5	The FAST process	a. Four-chamber heart below the xiphoid process shows the pericardial cavity, b. Section of the crypt of the liver and kidney, c. Section of the crypt of the spleen and kidney, d. Section of the pelvic cavity of the lower abdomen, e. Section of the bilateral chest cavity, etc

The theoretical training content is shown in Table 1.

At the beginning of the practical training, a handheld ultrasound was carried out under the guidance of the provincial hospital. The practical training content is practical in Table 2.

The content of the theory test is based on knowledge obtained in class. After each skill training, 5 specific sections are required to be completed within 5 min, and the practical results are determined from the aspects of image quality, technique, special parts annotation and proficiency [3]. Two teachers graded and averaged the scores. Doctors who passed the theoretical and practical training with the training certificate were required to provide 5 cases per month. After the completion of the training, the doctors were required to complete the questionnaire to investigate the corresponding problems in the “Feedback Evaluation Form of Taishun Ultrasound Ability Improvement Training Course”. The training satisfaction levels ranged from 0 to 10 points.

**Fig. 1:**
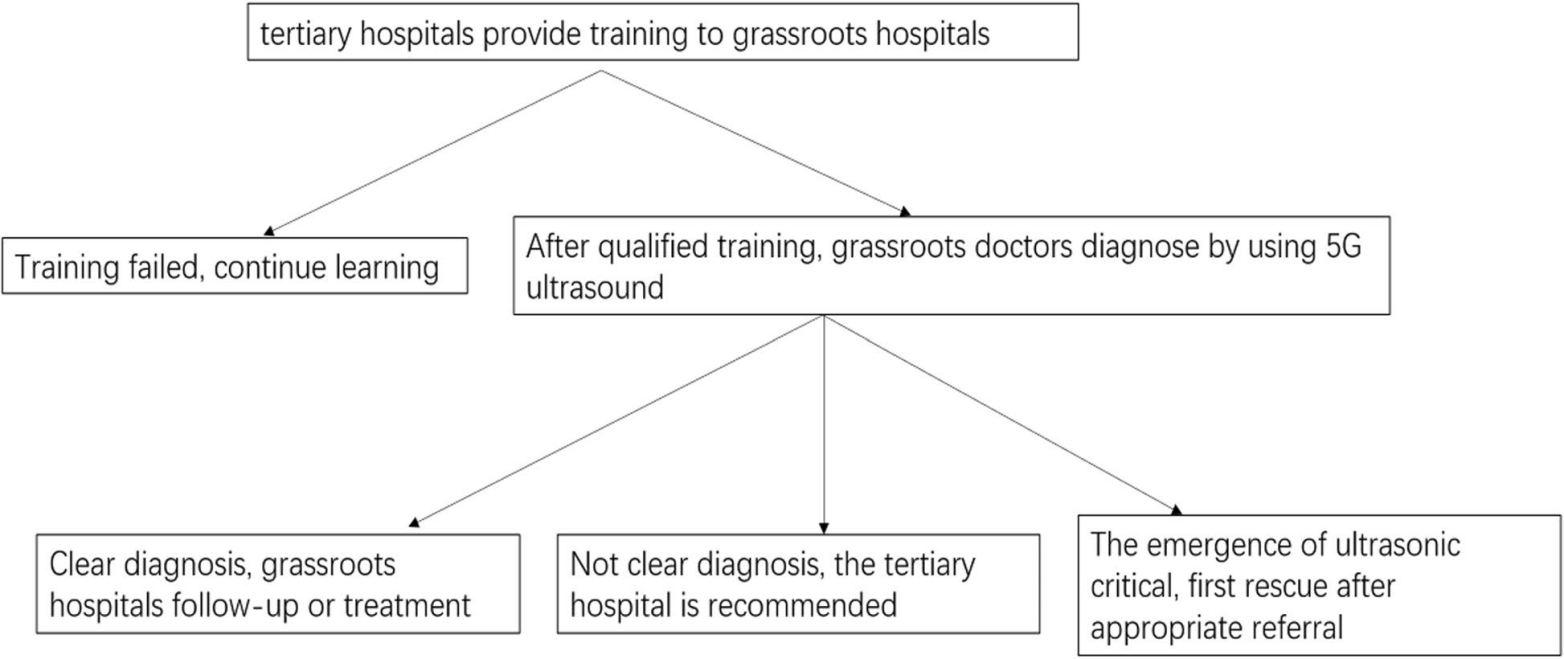
Flow chart of remote consultation

The “stroke” remote ultrasonic and remote platform has the function of real-time remote transmission of ultrasonic images and two-way voice calls. The image can be stored on a remote platform for consultation with experts to comment and analyse. All materials were uploaded to the database to provide a platform for learning and exchange. The flow chart of remote consultation is shown in Fig. 1 below.

### Statistics

SPSS 19.0 statistical software was used, and the enumeration data were expressed by quantity and percentage. Measurement data are described as the means ± standard deviation (M ± SD), and a t test was used for comparisons between two independent samples. *P* < 0.05 was considered statistically significant.

## Results

### Practical assessment and theoretical training results

The training class involved 2 practical assessments and 1 theoretical assessment with a total score of 50 points for each assessment. The difference in scores between the two practical trainings was statistically significant. See Table 3 for relevant results.

**Table 3:** Practical and theoretical results

Practical Training 1	Practical Training 2	Theoretical results
25.44 ± 6.81	40.81 ± 8.15*	34.59 ± 10.97

^*^shows *P* < 0.05

### Feedback from trainees after the training

Forty-three questionnaires were sent out, and all questionnaires were collected after the training. Scores included 0–5 for dissatisfied, 6–7 for satisfied, and 8–10 for very satisfied. Satisfaction scores are shown in Table 4.

**Table 4:** Satisfaction Questionnaire

Satisfaction score (points)	Number of trainees (persons)
10	35
9	5
8	2
7	1
6	0
0–5	0

### Remote consultation cases

**Fig. 2:**
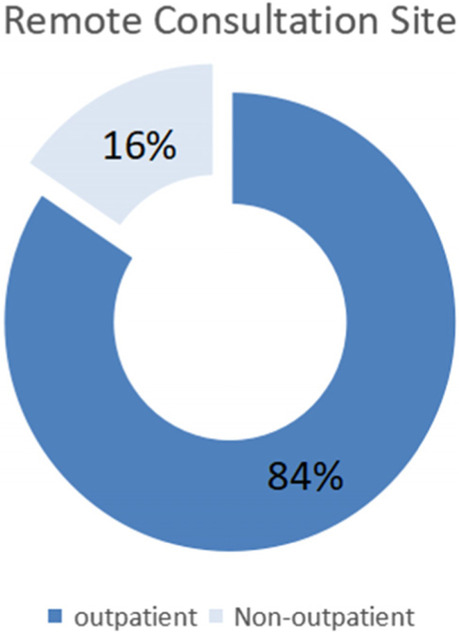
Proportion of remote consultation sites

**Fig. 3:**
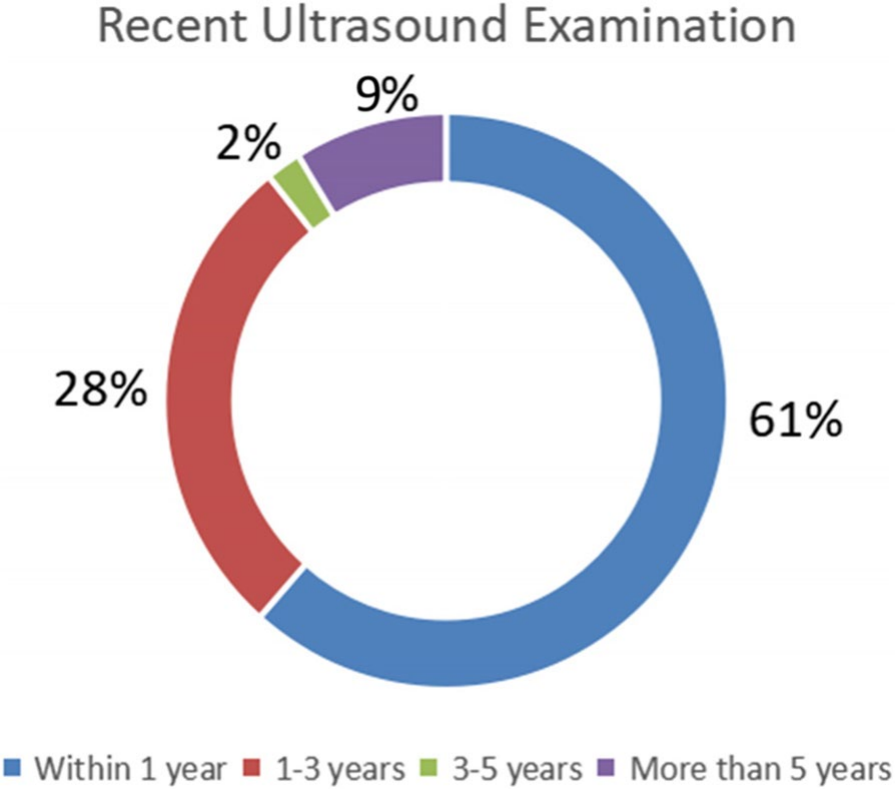
Proportion of the most recent ultrasound assessment for different time periods

**Fig. 4:**
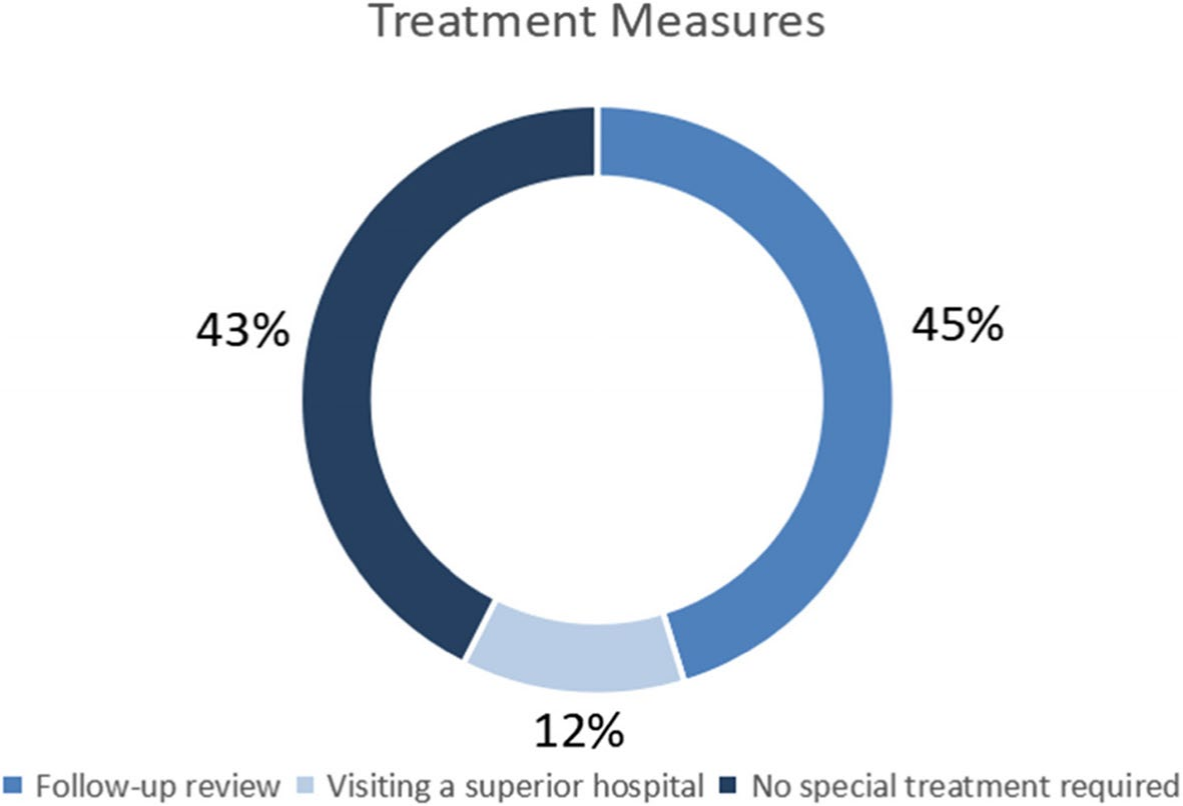
Proportions of different treatment measures

In total, there were 148 consultation cases, including 67 males and 81 females, who were aged 21 to 101 years old (62.40 ± 15.73). There were 47 cases of liver diseases (fatty liver, hepatic cyst, hepatic haemangioma, etc.), 16 cases of gallbladder diseases (cholecystitis, gallstones, polyps, etc.), and 29 cases of urinary diseases (urinary calculi, hydronephrosis, prostatic hyperplasia, bladder wall coarser, etc.). Other relevant information is shown in the following figures, including Fig. 2 showing the proportion of remote consultation sites, Fig. 3 showing the proportion of the most recent ultrasound assessment at different time periods and Fig. 4 showing the proportions of different treatment measures.

## Discussion

The training was initiated by the Department of Ultrasound of Zhejiang Provincial People's Hospital and the Vocational Training Center for Ultrasound Medicine of Zhejiang Province, based on the Taishun County People's Hospital and 12 affiliated community health hospitals. The establishment of a 5G ultrasound remote consultation mode between provincial-level Grade A hospitals and grassroots hospitals, relying on the information systems of counties, towns, villages and first aid to link ultrasonic comprehensive service stations, can better serve grassroots patients [4, 5]. With planned training, the instructors understood the characteristics and requirements of local medicine in advance and had the teaching goal of "how to use handheld ultrasound quickly and accurately". The theoretical training focused on the basic principles and usage of a handheld ultrasound as well as the most practical and most urgently needed ultrasonic knowledge for grassroots doctors. Practical training of students was divided into small groups for "hands-on teaching". After passing the theoretical and practical assessment, the trainees' practical scores were significantly improved. After obtaining training certificates, they carried out the diagnosis and treatment work in their respective areas. Through the feedback of students and their suggestions on our training course, we found the following: (1) Students believe that this type of training is very practical, but the class is compact and knowledge that needs to be retained is huge. They hope that the class can be extended to provide enough time for retention. (2) The theoretical courses are difficult; thus, the principles of ultrasound physics can be appropriately omitted. For general practitioners who have no ultrasound foundation, they prefer to have quick and simple introductory courses. (3) An instructor responsibility system was implemented in training, with one tutor leading 2–3 students. Small class teaching was optimized, and there was enough training time to refine each aspect and pass the test one by one participant.

Thus far, we have completed a total of 148 consultations through the remote ultrasound "visualization apparatus". Abdominal ultrasound was the main method, mainly for fatty liver, liver cysts, gallstones, and kidney stones. Due to the weak health awareness and lack of medical resources at the grassroots level, some patients have not undergone ultrasound examination for more than 5 years. For diseases found in the examination process, such as fatty liver, early detection can help us recommend diet and exercises to avoid abnormal liver malfunction and even liver failure caused by the aggravation of fatty liver. For some diseases with obvious pain symptoms, such as stone diseases, ultrasound can help provide the appropriate diagnosis and treatment recommendations. Patients can receive early detection and treatment, reduced duration of pain, and improved quality of life. If there is no 5G ultrasound and patients are not willing to or are unwilling to accept provincal hospital treatment, grassroots doctors can only rely on experience and simple clinical measures, such as “visual, touch, knocking, listening” and other measures, for symptomatic treatment. The probability of missed diagnoses and misdiagnosis will increase. If the diagnosis direction is erroneous, patients cannot receive effective treatment, and treatment can be delayed.

“Visualization apparatus” is a newly developed technique that differs from static imaging, such as radiation and electrocardiography, and real-time dynamic scanning is very important. The quality of images is largely determined by the manipulation, and clearer and more comprehensive images will lead to a more accurate diagnosis. Provincial experts can observe the size, shape, peripheral tissue relationship and blood flow signals of lesions in real time through a remote consultation system and guide the operation techniques of grassroots doctors [6, 7]. On the basis of voice and video, experts can not only communicate with patients to ask for a brief history but also make simple visual examinations, such as body mass, determining the location of pain and other relevant auxiliary information. In addition, through real-time explanation and guidance from experts, grassroots doctors can greatly improve their diagnostic coincidence rates.

This process is also conducive to the expansion of their own diagnostic ideas and improving the accuracy of diagnosis [8], and the reputations of grassroots doctors are improving to attract patients to seek medical treatment. Common and suitable cases can be solved in grassroots hospitals, which are conducive to the development of hierarchical diagnosis and treatment in grassroots hospitals and relieves the pressure from patients seeking treatment in large hospitals and the associated pressure on doctors.

There are also relevant literature reports that 5G remote ultrasound can be used to examine COVID-19 patients and transmit images to experts outside the isolation area, which can not only reduce the contact of medical staff but also provide better diagnosis and treatment resources, with good clinical and scientific research value.

Limitations of 5G ultrasound are that the operation level of grassroots doctors is not the same, and the flexibility of ultrasound scans is relatively large. If grassroots doctors do not scan the standard section or observe the best section of the lesion, remote experts cannot reasonably judge the condition. In the future, we will regularly organize experts to carry out theoretical and teaching training at the grassroots level to improve operation skills and clinical diagnosis. At the same time, we can conduct online learning exchange meetings with grassroots doctors to determine the local frequently occurring diseases, provide diagnosis and treatment experience, and deepen the reform and development of grassroots remote ultrasound medical treatment.

## Conclusion

In summary, ultrasonic remote consultation can not only help grassroots patients and realize hierarchical diagnosis and treatment but also promote the improvement of the clinical level and ultrasonic skills of grassroots doctors [9, 10]. With the development of 5G networks and telemedicine on the right track, remote ultrasound, as a “visual apparatus”, has been routinely carried out in grassroots hospitals. The effect will be on all aspects: mutual benefit between doctors and patients, improvement of grassroots infrastructure conditions, and improvements in disease prevention, treatment and health management capabilities. All of these form a grassroots medical community system and promote overall healthy villages. At the same time, in the current epidemic of COVID-19, we can use remote ultrasound and robots to examine infected people, reduce transmission routes and avoid infection of health care workers, which can also be used in research studies.

## Data Availability

All data generated or analysed during this study are included in this published article, The datasets generated and analysed during the current study are not publicly available due to The data may be applied to other relevant studies but are available from the corresponding author on reasonable request. If you want to obtain relevant data and materials, please contact email:wangjing@hmc.edu.cn. Thank you for your understanding.
